# Infectivity, effects on helper viruses and whitefly transmission of the deltasatellites associated with sweepoviruses (genus *Begomovirus*, family *Geminiviridae*)

**DOI:** 10.1038/srep30204

**Published:** 2016-07-25

**Authors:** Ishtiaq Hassan, Anelise F. Orílio, Elvira Fiallo-Olivé, Rob W. Briddon, Jesús Navas-Castillo

**Affiliations:** 1 Instituto de Hortofruticultura Subtropical y Mediterránea “La Mayora”, Universidad de Málaga - Consejo Superior de Investigaciones Científicas (IHSM-UMA-CSIC), Estación Experimental “La Mayora”, 29750 Algarrobo-Costa, Málaga, Spain; 2 Agricultural Biotechnology Division, National Institute for Biotechnology and Genetic Engineering, Faisalabad, Pakistan; 3 Pakistan Institute of Engineering and Applied Sciences, Islamabad, Pakistan

## Abstract

Begomoviruses (family *Geminiviridae*) are whitefly-transmitted viruses with single-stranded DNA genomes that are frequently associated with DNA satellites. These satellites include non-coding satellites, for which the name deltasatellites has been proposed. Although the first deltasatellite was identified in the late 1990s, little is known about the effects they have on infections of their helper begomoviruses. Recently a group of deltasatellites were identified associated with sweepoviruses, a group of phylogenetically distinct begomoviruses that infect plants of the family Convolvulaceae including sweet potato. In this work, the deltasatellites associated with sweepoviruses are shown to be transreplicated and maintained in plants by the virus with which they were identified, sweet potato leaf curl virus (SPLCV). These deltasatellites were shown generally to reduce symptom severity of the virus infection by reducing virus DNA levels. Additionally they were shown to be maintained in plants, and reduce the symptoms induced by two Old World monopartite begomoviruses, tomato yellow leaf curl virus and tomato yellow leaf curl Sardinia virus. Finally one of the satellites was shown to be transmitted plant-to-plant in the presence of SPLCV by the whitefly vector of the virus, *Bemisia tabaci*, being the first time a deltasatellite has been shown to be insect transmitted.

Single-stranded (ss) DNA satellites have recently been identified in sweet potato (*Ipomoea batatas*) and *I. indica* in association with sweepoviruses^1^. Sweepoviruses are phylogenetically distinct viruses of the genus *Begomovirus* (family *Geminiviridae*)^2^,^3^,^4^. Begomoviruses are transmitted by the whitefly *Bemisia tabaci* (Hemiptera: Aleyrodidae) and have genomes that consist of either one or two components^5^,^6^. The genomes of begomoviruses native to the New World (NW) are mostly bipartite, whereas most Old World (OW) begomoviruses are monopartite and associate with additional ssDNA molecules; alphasatellites and betasatellites. In the NW only alphasatellites have been identified^7^,^8^.

OW monopartite begomoviruses encode 6 genes including, in the complementary-sense, the replication-associated protein (Rep); a rolling-circle replication (RCR) initiator protein. Between the bidirectional coding sequences lies an intergenic region (IR) containing the origin (*ori*) of virion-strand DNA replication. The *ori* consists of a predicted hairpin structure, containing the nonanucleotide motif TAATATTAC, and short, repeated motifs (iterons). Begomoviruses replicate their genomes by RCR^9^. This is initiated by Rep, which recognizes the iterons and nicks the virion-strand in the nonanucleotide to initiate RCR. The sequence specificity for interaction with iterons ensures that the Rep of one virus species cannot generally initiate replication at the *ori* of a distinct species.

Betasatellites (~1360 nt) are circular, ssDNA satellites that depend upon a helper begomovirus for replication, movement in and between plants^10^,^11^. They encode a single gene (βC1), a sequence rich in adenine (A-rich) and a sequence conserved among all betasatellites (the satellite conserved region [SCR])^12^. Betasatellites rely upon helper virus-encoded Rep to initiate RCR. The SCR contains a predicted stem-loop structure with the nonanucleotide sequence TAATATTAC^12^,^13^. Betasatellites lack the iterons of their helper begomoviruses and Rep is believed to instead recognize iteron-like sequences residing between the A-rich and SCR sequences^14^,^15^.

The first DNA satellite identified was in association with the OW monopartite begomovirus tomato leaf curl virus (ToLCV)^16^. ToLCV-associated satellite (ToLCV-sat) is approximately one quarter (682 nt) the size of ToLCV, non-coding, has an A-rich region and a sequence related to the betasatellite SCR containing a hairpin structure with a TAATATTAC nonanucleotide sequence. This suggests that ToLCV-sat arose as a defective betasatellite^10^. Unlike betasatellites, ToLCV-sat encodes a sequence identical to the iterons of ToLCV on a second hairpin structure, lying between the A-rich and SCR-like sequences, which plays a role in the interaction with ToLCV Rep^16^,^17^. ToLCV-sat can be trans-replicated and maintained *in planta* by geminiviruses which do not share iterons with ToLCV and Rep-iteron interaction was shown to not strictly be required for initiation of replication^17^.

The sweepovirus-associated satellites (SAS) resemble ToLCV-sat in all respects including encoding an iteron sequence of their helper begomovirus(es) in a second stem-loop structure^1^. Several ToLCV-sat-like satellites have been identified and the collective name “deltasatellites” has been proposed for them^1^,^18^. Only for ToLCV-sat has transreplication been investigated and for none have the effects of the satellite on virus infection been investigated.

The study described here has investigated the interaction of the SAS with the associated sweepovirus, sweet potato leaf curl virus (SPLCV), and two heterologous begomoviruses. This showed that SAS can be trans-replicated and maintained in plants by various begomoviruses and that the satellites may ameliorate symptoms and reduced virus DNA levels. Additionally one SAS was shown to be transmitted by *B. tabaci* in the presence of SPLCV, being the first deltasatellite shown to be insect transmissible.

## Results

### Infectivity of the satellites in the presence of SPLCV in *N. benthamiana*

**Figure 1:**
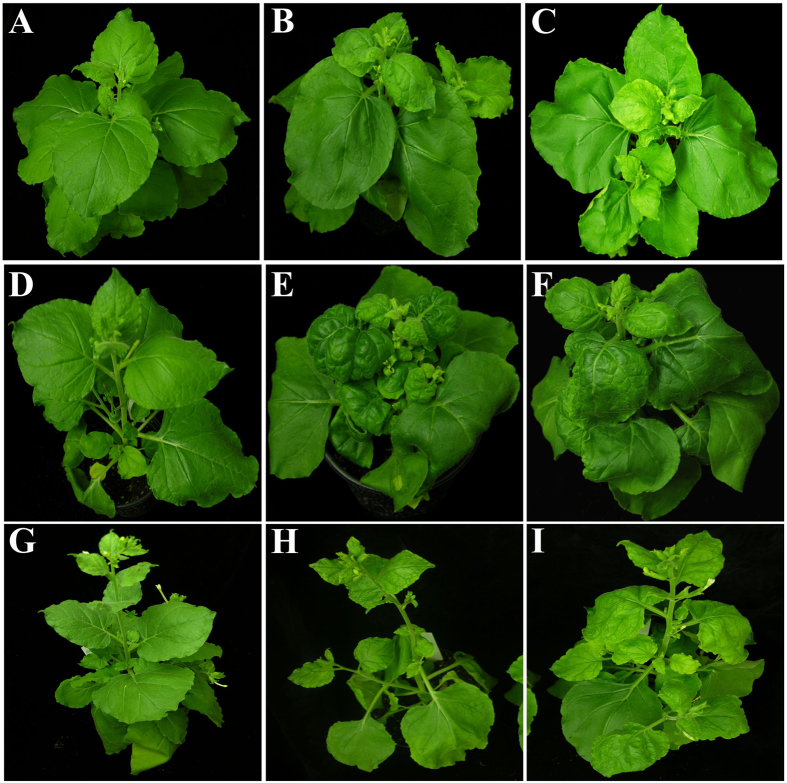
Symptoms induced by begomoviruses in the presence of satellites in *N. benthamiana* plants. The plants shown were either non-inoculated (**A**,**D**,**G**) or inoculated with SPLCV (**B**), SPLCV and DIM-SBG51 (**C**), TYLCV (**E**), TYLCV and DIM-SBG51 (**F**), TYLCSV (**H**) or TYLCSV and DIM-SBG51 (**I**). The photographs were taken at 22 dpi.

**Figure 2:**
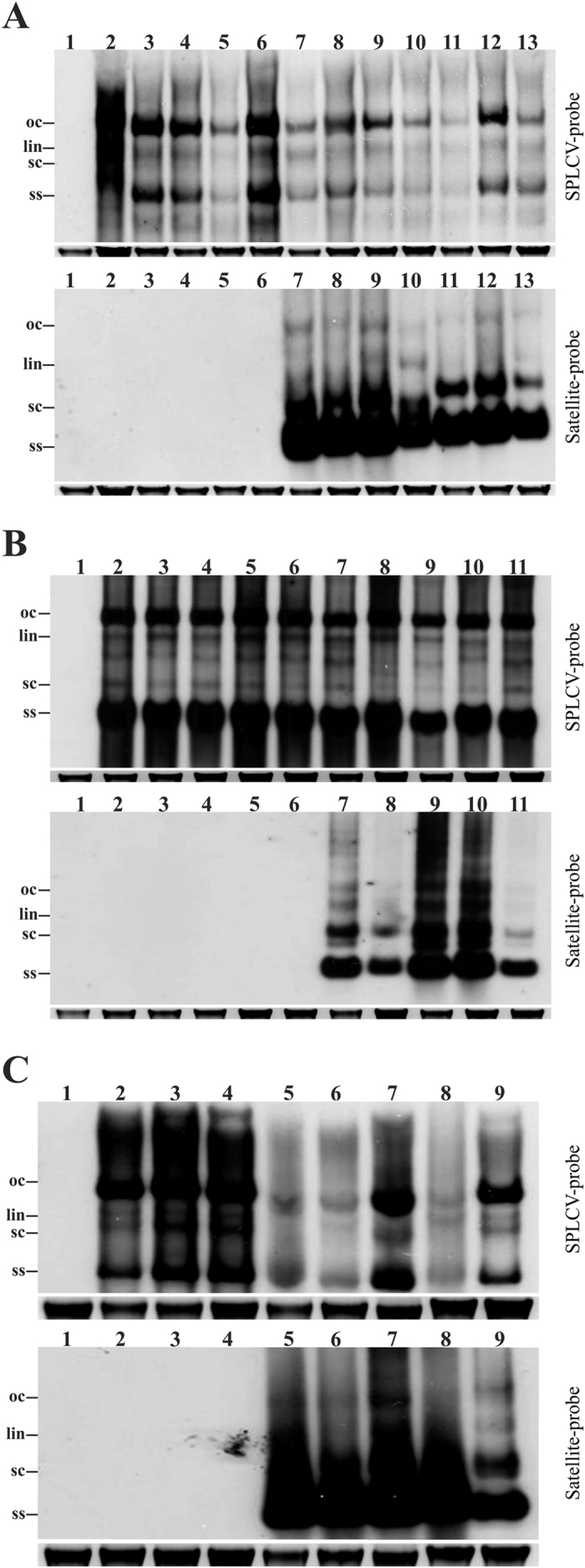
Southern blot analysis of DNA samples extracted from plants infected with SPLCV and satellites by either *Agrobacterium*-mediated inoculation of *N. benthamiana* (**A**) *Agrobacterium*-mediated inoculation of *I. setosa* (**B**) or insect transmission to *I. setosa* (**C**). Blots were probed for the presence of SPLCV (upper panel) and SBG51 (lower panel) in each case. (**A**) The DNA extracts were from a healthy non-inoculated *N. benthamiana* (lane 1) and *N. benthamiana* plants inoculated with SPLCV (lanes 2–6), SPLCV/DIM-SBG51 (lanes 7–10) and SPLCV/DIM-SBG59 (lanes 11–13). (**B**) The DNA extracts were from a healthy non-inoculated *I. setosa* (lane 1) and *I. setosa* plants inoculated with SPLCV (lanes 2–6) and SPLCV/DIM-SBG51 (lanes 7–11). (**C**) The DNA extracts were from a healthy non-inoculated *I. setosa* plant (lane 1) and *I. setosa* plants inoculated with *B. tabaci* insects fed on a SPLCV/SBG51 infected plant (lanes 2–9). The positions of viral single stranded (ss) super-coiled (sc), linear (lin) and open-circular (oc) DNAs are indicated. The ethidium bromide-stained genomic DNA band on the gel is shown below the Southern blot in each case to confirm equal loading. DNA was extracted at 20 dpi and 6 μg of total DNA were loaded in each case.

*Agrobacterium*-mediated inoculation (agroinoculation) of SPLCV to *N. benthamiana* resulted in the majority of plants becoming symptomatic (18 out of 20 plants inoculated; Table 1). Initial symptoms of infection, consisting of leaf yellowing and mild leaf curling appeared at 16 dpi. At approx. 22 dpi plants showed foliar yellowing, downward leaf curling, crumpling and stunting of plants (Fig. 1; Table 1). *N. benthamiana* plants inoculated with SPLCV in association with one of the satellites showed the same symptoms irrespective of the satellite used (Fig. 1, Table 1). The symptoms induced by SPLCV in the presence of satellites did not differ significantly from the symptoms induced by the virus in plants in the absence of the satellites. Plants inoculated with only DIM-SBG51 did not develop symptoms and the satellite could not be detected in leaves distal to the inoculation site (results not shown). Southern/dot-blot/PCR analysis showed that the satellites were maintained by SPLCV in all virus infected plants (Fig. 2, Table 1) and the infectivity of the virus to *N. benthamiana* was not affected by the satellites. A Southern blot of *N. benthamiana* plants co-inoculated with SPLCV and either DIM-SBG51 or DIM-SBG59 showed the DNA forms for virus and satellites typical of RCR (Fig. 2A). However, the blot also showed an apparent reduction of the amount of viral DNA for plants infected with the virus in the presence of either of the satellites in comparison to plants infected with only the virus. A densitometric analysis using ImagJ indicated the viral DNA levels to be approx. 40% lower in the presence of the satellites (Supplementary Fig. 1A).

### Infectivity of satellites in the presence of SPLCV in *I. setosa* and *I. nil*

**Figure 3:**
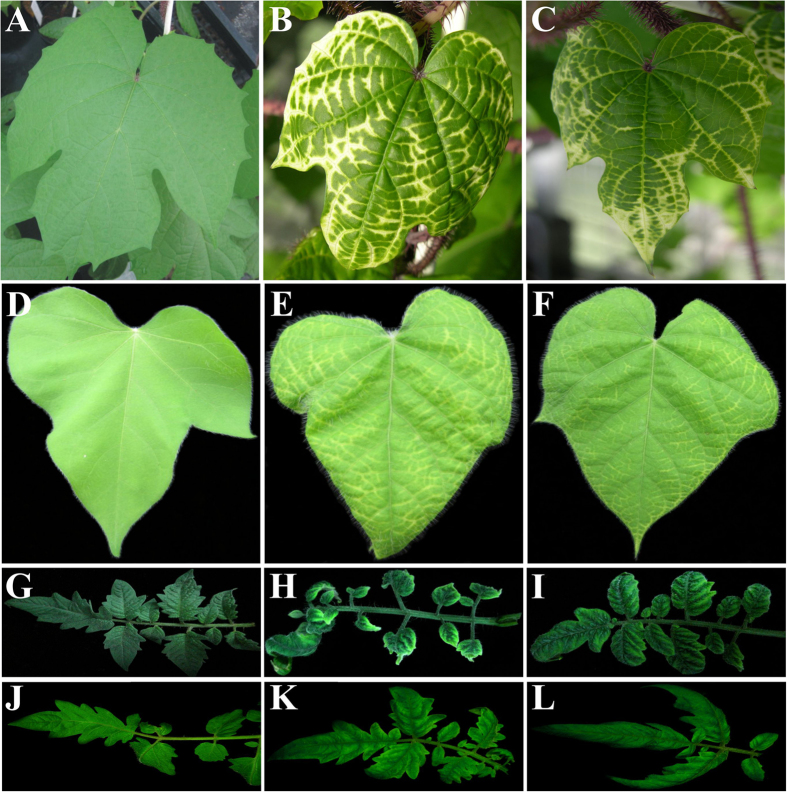
Foliar symptoms induced by infections of plants with begomoviruses and satellites. A leaf of a non-inoculated *I. setosa* plant (**A**) and leaves of *I. setosa* plants inoculated with SPLCV (**B**) or SPLCV and DIM-SBG51 (**C**). A leaf of a non-inoculated *I. nil* plant (**D**) and leaves of *I. nil* plants inoculated with SPLCV (**E**) and SPLCV and DIM-SBG51 (**F**). Leaves of non-inoculated tomato plants (**G**,**J**) and leaves of plants inoculated with TYLCV (**H**), TYLCV and DIM-SBG51 (**I**), TYLCSV (**K**) or TYLCSV and DIM-SBG51 (**L**). The photographs of *I. setosa* and *I. nil* were taken at 35 dpi whereas the photographs of tomato were taken at 40 dpi.

Agroinoculation of *I. setosa* and *I. nil* with SPLCV resulted in most plants becoming symptomatic (Table 1). *I. setosa* and *I. nil* differed in the latent period for symptoms appearing; 18 days and 17 days, respectively. Both plant species initially showed some vein yellowing and leaf curling/crumpling which reached a peak at approx. 35 dpi. For *I. setosa* the vein yellowing was more pronounced than for *I. nil* (Fig. 3). The vein yellowing initiated at the leaf margins and progressed towards the main vein, although there was no yellowing along the main vein. Infected plants were stunted relative to non-inoculated plants, with fewer leaves and a reduced leaf size, which was particularly evident for *I. nil*. Overall the symptoms induced in both *I. setosa* and *I. nil* by inoculation with SPLCV in the presence of the satellites were qualitatively similar to plants inoculated with virus alone and the latent period was extended by one to two days (Table 1) with milder symptoms (results not shown). Overall plants infected with virus and satellite were less stunted and had more and larger leaves than plants infected with only virus. Two inoculated *I. setosa* plants showed no symptoms, although SPLCV was present in both plants and the satellite in one (Table 1). In both *I. setosa* and *I. nil*, SBG57 was less infectious in the presence of SPLCV than the other satellites (Table 1). Band intensity analysis for the Southern-blot of SPLCV and SBG51 infected *I. setosa* plants showed no significant difference in virus titre in the presence/absence of the satellite (Supplementary Fig. 1B). However, a plot of virus DNA levels against satellite DNA levels suggested that there was an inverse relationship between the two – lower virus levels at higher satellite levels (R^2^ = 0.8094, Supplementary Fig. 1C).

### Infectivity of the satellites in the presence of SPLCV in sweet potato

No sweet potato plants developed symptoms following agroinoculation with SPLCV or SPLCV with either DIM-SBG51 or DIM-SBG57. However, the presence of SPLCV and satellites was detected in a small number of plants (Table 1). Poor infectivity and a long latent period for agroinoculation of sweet potato have been noted previously^4^. Instead grafting was used to investigate the effects of satellites on virus infection in sweet potato. Grafting with scions of *I. setosa* plants infected with SPLCV/SBG51 by agroinoculation to root stocks of healthy sweet potato plants also did not result in symptoms. However, the presence of virus/satellite was detected in most of the inoculated plants by PCR and Southern hybridization (Table 2). Graft inoculation of *I. setosa* plants resulted in symptoms consisting of severe leaf curling, vein yellowing and deformed growth. These symptoms were indistinguishable from the symptoms induced in *I. setosa* plants by agroinoculation of the virus. In the two graft-inoculated, symptomatic *I. setosa* plants the presence of SPLCV and satellite was confirmed by PCR and hybridization (results not shown). However, for SPLCV/satellite inoculated Promesa plants, SPLCV was detected in three out of four plants and only two also contained the satellite. Of four graft-inoculated Beauregard plants, three were positive for both SPLCV and satellite (Table 2). Hybridization showed the DNA levels of satellite and SPLCV were low in sweet potato in comparison to infected *I. setosa* plants. SPLCV and satellite were detected in more Promesa plants than Beauregard plants. A long exposure time for the blots was required to see the signal for satellite in graft-inoculated Beauregard plants (Supplementary Fig. 2A).

### Transmission of satellite SBG51 by *Bemisia tabaci* in the presence of SPLCV

Transmission of SBG51 was assessed using *B. tabaci* (Mediterranean species) insects fed for 48 hrs on an *I. setosa* plant infected with SPLCV and SBG51 by agroinoculation before the insects were transferred to healthy *I. nil* and *I. setosa* plants for 48 hrs (50 insects per plant). In the first experiment eight out of twenty *I. nil* plants developed symptoms. Symptoms appeared at 17 dpi and by 35 dpi the plants were showing severe leaf curling, vein yellowing and stunting, as described for plants infected by agroinoculation. Dot-blot and PCR showed SPLCV, but not the satellite, to be present in symptomatic plants and two non-symptomatic plants (Table 2). For 15 out of 30 whitefly-inoculated *I. setosa* plants symptoms appeared at 21 dpi and plants developed symptoms indistinguishable from those exhibited by the plants used as the virus source, consisting of yellowing, mild leaf curling and stunting. Dot-blot hybridization and PCR showed the presence of SPLCV in 22 plants and SBG51 in 19 (Table 2). The non-symptomatic *I. setosa* plants were negative for both the virus and satellite (Table 2). Southern hybridization of six randomly selected SPLCV/SBG51 infected *I. setosa* plants and the three plants which contained only SPLCV following insect transmission showed the DNA forms typical of virus and satellite replication (Fig. 2C). Although the viral DNA levels in plants containing SPLCV and SBG51 were quite variable, overall the virus DNA levels were lower than in plants harbouring only virus. Analysis of band intensities showed that SPLCV DNA levels in plants co-infected with the satellites were ~40% of those for plants infected with the virus alone (Supplementary Fig. 1D).

### Effects of sweepovirus-associated satellites on TYLCV and TYLCSV infections of *N. benthamiana* and tomato

**Figure 4:**
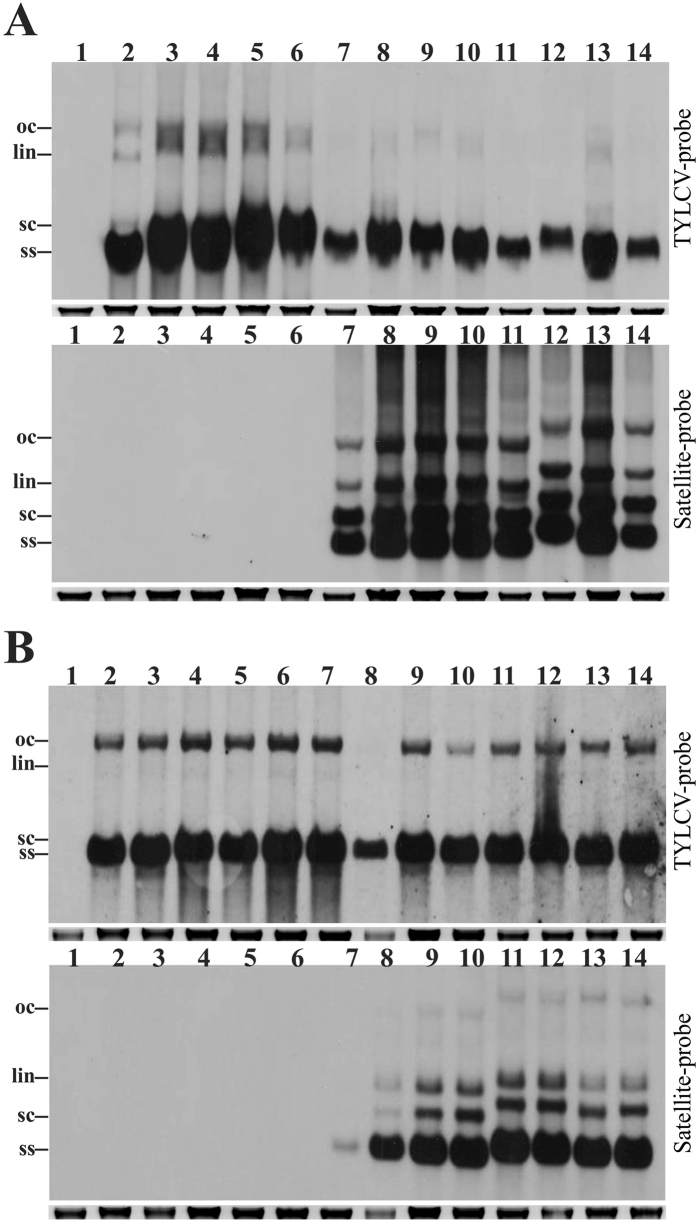
Southern blot analysis of DNA samples extracted from *N. benthamiana* (**A**) and tomato (**B**) plants infected with TYLCV and satellites by *Agrobacterium*-mediated inoculation. Blots were probed for the presence of TYLCV (upper panel) and SBG51 (lower panel) in each case. (**A**) The DNA samples were extracted from a non-inoculated *N. benthamiana* plant (lane 1) and *N. benthamiana* plants inoculated with TYLCV (lanes 2–6), TYLCV and DIM-SBG51 (lanes 7–11) and TYLCV and DIM-SBG59 (lanes12–14). (**B**) The DNA samples were extracted from a non-inoculated tomato plant (lane 1) and tomato plants inoculated with TYLCV (lanes 2–6), TYLCV and DIM-SBG51 (lanes 7–10) and TYLCV and DIM-SBG59 (lanes 11–14). DNA was extracted from plant samples at 20 dpi and in each lane 6 μg of total DNA was loaded. The positions of viral DNA forms are labelled as single-stranded (ss), supercoiled (sc), linear (lin) and open circular (oc). The ethidium stained genomic DNA band of the gel is shown below the Southern blot in each case to confirm equal loading.

*N. benthamiana* plants inoculated with TYLCV started showing initial symptoms of infection at 15 dpi consisting of leaf yellowing at the margins of young leaves and mild leaf curling. Subsequently, symptoms became more severe consisting of severe downward leaf curling, leaf crumpling, yellowing and stunting (Table 1; Fig. 1). The internodes of young, newly developing leaves were reduced and the leaves were cup-shaped. Non-inoculated plants (Fig. 1) and plants inoculated with only DIM-SBG51 were non-symptomatic and satellite could not be detected in young leaves (results not shown). *N. benthamiana* plants inoculated with TYLCV and either DIM-SBG51 or DIM-SBG59 exhibited symptoms that were overall milder than plants inoculated with only TYLCV and the latent period was longer (Table 1). The plants initially exhibited leaf yellowing at 17 dpi, which started from the leaf margins. From approx. 22 dpi onwards, young newly developing leaves showed mild downward leaf curling. Overall plants co-infected with the satellites showed less leaf curling and more yellowing than plants infected with the virus alone (Fig. 1). Dot-blot hybridization confirmed the presence of the satellites as well as TYLCV in all co-inoculated plants. Southern hybridization detected the characteristic DNA forms typical of begomovirus and satellite replication (Fig. 4A). The titre of TYLCV was higher in *N. benthamiana* plants infected with only TYLCV than in plants co-infected with either DIM-SBG51 or DIM-SBG59. Analysis of band intensities indicated that TYLCV levels in plants co-infected with the satellites were ~40% those for plants infected with only the virus (Supplementary Fig. 1E).

TYLCV was highly infectious to tomato cv. Moneymaker plants by agroinoculation. Symptoms of infection started with leaf yellowing at 17 dpi. The symptoms then became more severe showing severe leaf yellowing (particularly along the leaf margins and interveinal tissues), severe leaf curling, leaf rolling, a reduced leaflet size and stunting (Table 1; Fig. 3). Non-inoculated tomato plants kept under the same conditions remained symptomless. Tomato plants co-inoculated with TYLCV and either DIM-SBG51 or DIM-SBG59 developed symptoms similar to plants inoculated with only TYLCV. The symptoms appeared at 18 dpi, possibly slightly delayed over plants inoculated with only TYLCV, and consisted of mild leaf yellowing (along the leaf margins), mild leaf curling and stunting (Fig. 3). Leaflets were not significantly reduced in size compared to healthy plants. TYLCV was detected in all inoculated plants by dot-blot hybridization. For plants co-inoculated with DIM-SBG51 the satellite was detected in all inoculated plants whereas with DIM-SBG59, the satellite was found in 3 out of 4 plants (Table 1). Southern hybridization showed the accumulation of both TYLCV and satellite DNA in tomato plants (Fig. 4B). Analysis of band intensities suggested that the satellites did not significantly affect the level of the virus (Supplementary Fig. 1F).

All TYLCSV inoculated *N. benthamiana* plants developed symptoms at 16 dpi (Table 1) consisting of yellowing at the margins of young leaves, which developed into mild upward and downward leaf curling, and plants were stunted (Fig. 1). *N. benthamiana* plants co-inoculated with TYLCSV and DIM-SBG51 or DIM-SBG59 developed symptoms that were similar to the symptoms of plants infected with only the virus but with less intense foliar yellowing and possibly a slight extension in the latent period (Fig. 1; Table 1).

Tomato plants inoculated with TYLCSV showed the first symptoms of infection at 19 dpi and all plants ultimately became symptomatic. The symptoms started with yellowing of the youngest leaves and subsequently downward leaf curling. Infected plants were shorter in stature than the healthy control plants. For tomato plants co-inoculated with virus and either satellite, the symptoms were qualitatively the same as plants infected with only virus. However, plants infected with TYLCSV and satellite showed less intense foliar yellowing and only mild leaf curling (Fig. 3). In contrast to tomato plants inoculated with only virus, for which all plants became symptomatic, not all plants ultimately showed symptoms when inoculated with virus and either of the satellites (Table 1). Non-inoculated *N. benthamiana* and tomato plants remained non-symptomatic (Table 1).

In all plants inoculated with TYLCSV only, and all *N. benthamiana* plants co-inoculated with TYLCSV and either of the satellites, virus was detected by dot-blot hybridization. The satellite and virus was also detected in tomato plants co-inoculated with TYLCSV and either of the satellites. However, for DIM-SBG51 only one of the three symptomatic plants showed the presence of the satellite whereas for DIM-SBG59 both of the symptomatic plants showed the presence of the satellite (Table 1). Southern hybridization showed a lower titer of TYLCSV in *N. benthamiana* plants co-infected with either DIM-SBG51 or DIM-SBG59 compared to plants infected with only TYLCSV (Supplementary Fig. 2B). An ImagJ quantification showed virus titer for TYLCSV/DIM-SBG51 and TYLCSV/DIM-SBG59 infected plants to be ~40% and ~60%, respectively, that in plants infected with TYLCSV alone (Supplementary Fig. 1G).

## Discussion

Inoculation of *N. benthamiana* plants with only SBG51 did not lead to symptoms and the satellite was not detected in young, newly emerging leaves. This is consistent with the idea that the satellite requires a helper virus for maintenance in plants^1^. When co-inoculated with the clone of SPLCV^4^, all plants infected with the virus also showed the presence of the satellite. This indicates that in *N. benthamiana* SPLCV efficiently moves, *in trans*, the satellites. It is also likely that SPLCV trans-replicated the satellites, since the satellites do not encode a RCR initiator protein. The presence of satellite DNA forms typical of RCR in Southern blots supports this hypothesis.

The reduction in virus DNA levels in the presence of the satellites is indicative of the satellites interfering with virus infection. The effect was, however, not so clear upon agroinoculation of *I. setosa*. The difference between insect transmission and agroinoculation is likely due to the amount of virus delivered. *B. tabaci* adults acquire minute amounts of virus and only a small fraction of this is inoculated to plants during a feed^19^. Whitefly transmission is thus a tight bottleneck and an initial infection would contain few virus copies that would be susceptible to interference by satellites. In contrast, agroinoculation delivers more virus with the possibility of cells being infected without the satellite, meaning interference by the satellite might be masked.

Sweepovirus infections are frequently latent (mildly or non-symptomatic)^2^, but the factors that lead to latency are unclear. For agroinoculated *I. setosa* and *I. nil* plants there was a clear extension in latent period for SPLCV infections in the presence of satellites which was not evident for *N. benthamiana*. This is likely due to *N. benthamiana* being a highly susceptible species due to it having a compromised RNA silencing response^20^. Possibly SPLCV is able to replicate and spread more effectively in *N. benthamiana*, masking the effects of the interference of the satellite on latent period and symptoms.

For betasatellites and alphasatellites several studies have investigated the effects of satellites on virus infections. Betasatellites encode a dominant pathogenicity determinant (βC1) that may enhance virus DNA levels in plants and exacerbate symptoms^10^,^11^. Alphasatellites may reduce virus DNA levels^21^ or reduce symptom severity by preferentially reducing betasatellite DNA levels^22^,^23^,^24^. For alphasatellites and defective interfering (DI) molecules the precise mechanism of interference with helper viruses, leading to symptom amelioration and reduced viral DNA levels, is unclear. It has been suggested that interference may be due to competition for cellular resources, such as the host DNA replication machinery^25^,^26^, and virus encoded factors, including those involved in virus movement in plants^27^,^28^. Additionally, for DI molecules, there may be competition for the helper virus-encoded Rep. Since the SAS rely on virus-encoded Rep for initiation of replication and virus-encoded factors for movement in plants, both of these mechanisms can be proposed to explain their interference with virus infections of plants.

Sweet potato is vegetatively propagated and the clone of SPLCV used in the study here has previously been shown to be transmissible by the Mediterranean species of the *B. tabaci* complex^4^. The results here show one of the SAS is transmissible in co-infection with SPLCV. This is the first demonstration of the insect transmission of a deltasatellite and indirectly demonstrates encapsidation of the satellite in the begomovirus CP. Geminivirus virions consist of only ssDNA and CP^29^,^30^,^31^,^32^ and the CP determines insect specificity for transmission^32^,^33^. DI molecules (~1400 bp) have been shown to be encapsidated in isometric (half geminate) particles^34^,^35^. Although betasatellites have been shown to be insect transmissible^10^ and encapsidated in helper virus CP^36^, the multiplicity of the particles has not been investigated. The nature of virus particles encapsidating deltasatellites (quarter unit-length DNAs) also remains unknown.

SBG51 was efficiently insect-transmitted from infected *I. setosa* plants to healthy *I. setosa* plants in the presence of SPLCV. This was not the case for insect transmission to *I. nil*, even though the same source plants and insect colony was used. It is unlikely that this is occurring due to an incompatibility between the satellite and *I. nil*, since by agroinoculation SBG51 was highly infectious in the presence of SPLCV to this species. SPLCV was less infectious to *I. nil* than to *I. setosa* using insect transmission, so possibly this has something to do with the inability of insects to transfer the satellites.

The results presented here additionally show that SAS can be trans-replicated and maintained in plants by heterologous begomoviruses. Both TYLCV and TYLCSV are not commonly associated with satellites, although one strain of TYLCV has been shown to associate with a betasatellite and an alphasatellite^22^,^37^. Also a TYLCV isolate not associated with satellites was shown to be capable of trans-replicating and maintaining a betasatellite in plants^38^. In this respect the SAS thus behave very much like ToLCV-sat and the betasatellites, apparently having little or no helper-virus specificity, and not like DNA-B components or DI-DNAs (derived from DNA B components) that show a high degree of helper-virus specificity. Since the SAS are efficiently transreplicated despite the absence of iterons it is likely that, as has been proposed for betasatellites^14^,^15^, SAS contain sequences that mimic iterons in the interaction with helper virus-encoded Rep^1^.

In *N. benthamiana*, the presence of a satellite ameliorated symptoms and retarded infections of both TYLCV and TYCSV which is consistent with the effects of alphasatellites and DI molecules^14^,^22^,^24^,^25^. There was a clear reduction in virus DNA levels, by as much as 60%, in the presence of the satellites. Virus levels in tomato also appeared reduced, although for TYLCV these were not statistically significant. The reason for this difference is unclear. TYLCV is the more aggressive virus, in some areas has displaced TYLCSV^39^ and has the ability to infect tomato varieties with resistance to other tomato-infecting begomoviruses^40^. Possibly TYLCV spreads more efficiently (rapidly) in tomato, masking interference by satellites.

Why SBG59 had a greater effect on the virus than SBG51 is unclear. SBG59 has a repeated sequence immediately 3’ of the nonanucleotide containing hairpin structure and it is tempting to speculate that this may be the reason for the difference. However, it is not possible to provide a mechanistic explanation as to why a repeated sequence should cause this effect and will require further investigation.

For both *N. benthamiana* and tomato the SAS had no effect on the numbers of plants that became infected with TYLCV. However, in tomato the inclusion of either satellite reduced the infectivity of TYLCSV and not all infected plants contained the satellite. This suggests that the satellites interfere with the infectivity of TYLCSV and that the host plays a part in this. However, a greater number of plants would need to be inoculated to accurately quantify this effect. It would also be of interest to see whether, at lower inoculum pressure, the effects of the satellites become more evident. At low virus inoculum doses, such as those delivered by whiteflies, the effects of the satellites on infectivity and symptoms may be greater.

Dry *et al*.^16^ did not address the effects of ToLCV-sat on virus infection or the selective advantage of maintenance of the satellite. For most betasatellites the βC1 protein provides a strong selective advantage for maintenance by a begomovirus. The βC1 protein is a suppressor of silencing and plays a part in virus movement in plants^11^,^41^,^42^. The situation for alphasatellites is less clear. Alphasatellites may attenuate symptoms by reducing the levels of betasatellite DNA without affecting virus DNA levels^22^,^23^. The SAS behave very much like the alphasatellites and virus-derived DI molecules, possibly by competing for cellular and virus-encoded resources/factors. It is likely that the SAS (and other deltasatellites) benefit the virus by reducing the damage caused, extending the life of the plant and the possibility of the virus being transmitted.

The use of DI molecules to engineer resistance against geminiviruses in model plants showed promise^43^,^44^ but studies in crops were less than encouraging^26^. Deltasatellites could possibly provide a reprieve for the “DNA interference” approach to geminivirus resistance. They are not virus-derived, reducing the chance of silencing, are non-coding and have the potential to provide broad-spectrum resistance.

The results presented here have shown that the SAS are infectious to plants in the presence of a helper begomovirus, attenuate virus symptoms and reduce virus DNA levels. The SAS have a relaxed specificity for their interactions with helper viruses and behave very much like DI molecules. The results also suggest that there is a helper virus and/or host element to the interaction of the virus with the satellites, although the precise basis for this is unclear.

## Methods

### Viruses and satellites used in the study

Constructs for the agroinoculation of SPLCV-[ES:Mal:BG30:06] (GenBank acc. no. EU839579), tomato yellow leaf curl virus (TYLCV, AJ489258) and tomato yellow leaf curl Sardinia virus (TYLCSV, Z25751) have been described^4^,^45^,^46^. Deltasatellites isolated from sweet potato originating from Málaga (Spain) and Lanzarote (Canary Islands, Spain) [SBG51 (FJ914390), SBG53 (FJ914393), SBG54 (FJ914394), SBG55 (FJ914395), SBG57 (FJ914397), SBG58 (FJ914398) and SBG59 (FJ914403)] were used^1^.

### Production of constructs for agroinoculation of satellites

Head-to-tail dimer constructs of the seven satellites mentioned above were produced essentially as described by Ferreira *et al*.^47^. Satellites were PCR amplified using primers MA1386/MA1387 (CCTTAGCTTCGCACGTAGCT/CTGCTTAGCGTAGCGGTTTGG). Amplified products of ~700 bp were purified, self-ligated and then amplified by rolling-circle amplification (RCA; TempliPhi DNA Amplification Kit, GE Healthcare, Little Chalfont, UK). RCA product was partially digested with *Pst*I. Satellite dimers (~1400 bp) were excised from agarose gels and ligated in pCAMBIA0380 (Cambia, Canberra, Australia). Constructs were confirmed by restriction with *Bam*HI, *Hin*dIII, and sequencing. Dimeric constructs will henceforth be identified by the prefix DIM.

### Agroinoculation

Dimeric constructs were transformed into *Agrobacterium tumefaciens* GV3101 by electroporation and a culture of transformed cells was incubated at 28 °C until an OD_600_ of 1 was achieved. The cells were harvested by centrifugation at 3000 g for 10 min and re-suspended in a solution containing 10 mM MES, 10 mM MgCl_2_ and 150 μM acetosyringone. For experiments where co-inoculation was required, equal volumes of the inocula were mixed. *N. benthamiana* and tomato (*Solanum lycopersicum*) cv. Moneymaker plants were inoculated at 4–5 leaf stage, *I. setosa* and *I. nil* at the cotyledon stage whereas sweet potato cuttings were inoculated at the 2–3 leaf stage. Plants were kept in an insect-proof growth chamber (25 °C day/20 °C night, 70% relative humidity, 16-h photoperiod at 250 μmol s^–1^ m^–2^ photosynthetically active radiation).

### Virus inoculation by grafting

Healthy *I. batata* (cv. Beauregard or Promesa) plants were used as root stock and were graft inoculated with scions from *I. setosa* plants infected by agroinoculation, as described previously^4^.

### Whitefly transmission

Non-viruliferous *B. tabaci* (Mediterranean species, formerly biotype Q) were reared on melon (*Cucumis melo* cv. ANC 42, La Mayora-CSIC seed bank) plants. Adult whiteflies (~3000) were released on infected *I. setosa* (source plant) and given an acquisition-access period of 48 hrs, after which the insects were transferred into clamp cages on healthy test plants. A single clamp cage, containing ~50 whiteflies, was applied to each healthy *I. setosa* or *I. nil* plant at the cotyledon stage. An inoculation-access period of 48 hrs was provided and insects were then killed with imidacloprid and pyriproxyfen. Plants were transferred to an insect-free greenhouse and examined daily for the appearance of symptoms.

### DNA extraction and virus/satellite detection

Total DNA was extracted from plants using the CTAB method^48^. The presence of begomovirus and satellites in agroinoculated, graft-inoculated and whitefly transmitted plants was confirmed by PCR and dot-blot hybridization. For dot-blot hybridization approximately equal volume (1 μl) of genomic DNA was loaded onto positively charged nylon membranes (Roche) and hybridized with specific, digioxigenin (DIG)-labeled probes. Probes for SPLCV (CP gene) and full length satellite were PCR amplified using specific primers described by Lozano *et al*.^3^ and MA1386/MA1387 respectively. A mixed TYLCSV and TYLCV probe was prepared by PCR amplifying the IRs using specific primer pairs MA14/MA15 and MA30/MA31^49^. The PCR products were mixed in equal proportions and labeled using a non-radioactive DIG DNA labeling kit (Roche). For Southern hybridization approximately equal amounts of genomic DNA (6 μg) were electrophoresed on 0.8% agarose gels and then transferred onto positively charged nylon membranes (Roche). The cross-linked DNA was hybridized with the specific probes. Hybridization was performed at 60 °C for 12–15 hours followed by high stringency washing at the same temperature. Hybridization signals were detected on X-ray film after treatment with CDP-Star (Roche) and the band intensities were quantified using ImagJ software (http://rsbweb.nig.gov.ij/). Begomoviruses and satellites were detected in plants by PCR using the primers used to make probes.

## Additional Information

**How to cite this article**: Hassan, I. *et al*. Infectivity, effects on helper viruses and whitefly transmission of the deltasatellites associated with sweepoviruses (genus *Begomovirus*, family *Geminiviridae*). *Sci. Rep.* **6**, 30204; doi: 10.1038/srep30204 (2016).

## Supplementary Material

Supplementary Information

## Figures and Tables

**Table 1:** Infectivity of the satellites and helper begomoviruses by agroinoculation.

Host Plant^1^	Inoculum^2^	No. symptomatic plants/No. inoculated plants	Detection		Latent period (dpi)	Symptoms^3^
			Virus	Satellite		
*N. benthamiana*	SPLCV	18/20	18/20	0/20	16	LY, LC, LCr, RG
	SPLCV/SBG51	14/15	14/15	14/15	16	LY, LC, LCr, RG
	SPLCV/SBG53	5/5	5/5	5/5	16	LY, LC, LCr, RG
	SPLCV/SBG54	5/5	5/5	5/5	16	LY, LC, LCr, RG
	SPLCV/SBG55	5/5	5/5	5/5	16	LY, LC, LCr, RG
	SPLCV/SBG57	8/10	8/10	8/10	16	LY, LC, LCr, RG
	SPLCV/SBG58	5/5	5/5	5/5	16	LY, LC, LCr, RG
	SPLCV/SBG59	10/10	10/10	10/10	16	LY, LC, LCr, RG
	SBG51	0/5	0/5	0/5	−	−
	none	0/20	0/20	0/20	−	−
*I. setosa*	SPLCV	13/15	13/15	0/15	18	SVY, LC, RG
	SPLCV+SBG51	9/10	10/10	10/10	20	SVY, LC, RG
	SPLCV+SBG55	4/4	4/4	4/4	20	SVY, LC, RG
	SPLCV+SBG57	4/7	5/7	4/7	20	SVY, LC, RG
	SPLCV+SBG59	3/4	3/4	3/4	20	SVY, LC, RG
	none	0/15	0/15	0/15	−	−
*I. nil*	SPLCV	5/5	5/5	0/5	17	VY, SDLC, RG
	SPLCV+SBG51	8/8	8/8	8/8	18	VY, SDLC, RG
	SPLCV+SBG57	4/5	4/5	3/5	18	VY, SDLC, RG
	none	0/5	0/5	0/5	−	−
SP Beauregard	SPLCV	0/5	1/5	0/5	−	−
	SPLCV+SBGG51	0/5	1/5	1/5	−	−
	SPLCV+SBG57	0/5	0/5	0/5	−	−
	none	0/5	0/5	0/5	−	−
SP Promesa	SPLCV	0/5	1/5	1/5	−	−
	SPLCV+SBG51	0/5	1/5	1/5	−	−
	SPLCV+SBG57	0/5	0/5	0/5	−	−
	none	0/5	0/5	0/5	−	−
*N. benthamiana*	TYLCV	10/10	10/10	0/10	15	SDLC, LCr, LY, IR, LC, ST
	TYLCV/SBG51	10/10	10/10	10/10	17	LY, MDLC
	TYLCV/SBG59	4/4	4/4	4/4	17	LY, MDLC
	SBG51	0/5	0/5	0/5	−	−
	none	0/10	0/10	0/10	−	−
Tomato	TYLCV	5/5	5/5	0/5	17	SLC, LR, SLY, S
	TYLCV/SBG51	5/5	5/5	5/5	18	MLC, LY, S
	TYLCV/SBG59	4/4	4/4	3/4	18	MLC, LY, S
	none	0/5	0/5	0/5	−	−
*N. benthamiana*	TYLCSV	10/10	10/10	0/10	16	YM, MULC, MDLC, S
	TYLCSV/SBG51	9/9	9/9	9/9	17	MLY, MULC, MDLC, S
	TYLCSV/SBG59	4/4	4/4	4/4	17	MLY, MULC, MDLC, S
	none	0/10	0/10	0/10	−	−
Tomato	TYLCSV	5/5	5/5	0/5	19	LY, DLC, S
	TYLCSV/SBG51	3/4	3/4	1/4	19	MLY, MDLC, S
	TYLCSV/SBG59	2/3	2/3	2/3	19	MLY, MDLC, S
	none	0/5	0/5	0/5	−	−

^1^SP, sweet potato.

^2^SPLCV, sweet potato leaf curl virus; TYLCSV, tomato yellow leaf curl Sardinia virus; TYLCV, tomato yellow leaf curl virus.

^3^DLC, downward leaf curling; IR, reduction in internode length; LCr, leaf crumpling; LC, leaf curling; LY, leaf yellowing; MDLC, mild downward leaf curling; MLY, mild leaf yellowing; MULC, mild upward leaf curling; RG, retarded growth; S, stunting; SDLC, severe downward leaf curling; SVY, severe vein yellowing; VY, vein yellowing; YM, yellowing of leaf margins.

**Table 2:** Infectivity of the SBG51 deltasatellite and SPLCV by grafting or whitefly (*B. tabaci*) transmission.

Inoculation method	Host Plant^1^	Inoculum^2^	No. symptomatic plants/No. inoculated plants	No. infected plants/No. inoculated plants		Latent period (dpi)	Symptoms^3^
				Virus	Satellite		
Grafting	*I. setosa*	SPLCV+SBG51	2/2	2/2	2/2	20	SVY, LC, RG
	SP Beauregard	SPLCV+SBG51	0/4	3/4	3/4	−	−
	SP Promesa	SPLCV+SBG51	0/4	3/4	2/4	−	−
Insect	*I. nil*	SPLCV+SBG51	8/20	10/20	0/20	17	VY, SDLC, RG
	*I. setosa*	SPLCV+SBG51	15/30	22/30	19/30	20	SVY, LC, RG

^1^SP, sweet potato.

^2^SPLCV, sweet potato leaf curl virus.

^3^LC, leaf curling; RG, retarded growth; SDLC, severe downward leaf curling; SVY, severe vein yellowing, VY, vein yellowing.
