# Supplementary material for: Infectivity, effects on helper viruses and whitefly transmission of the deltasatellites associated with sweepoviruses (genus Begomovirus, family Geminiviridae)
Source: Sci Rep. 2016 Jul 25;6:30204. doi: 10.1038/srep30204 (PMC4958995; doi:10.1038/srep30204)
Supplement: Supplementary Information [file srep30204-s1.pdf]

Supplementary information

Infectivity, effects on helper viruses and whitefly transmission of the deltasatellites associated with sweepoviruses (genus *Begomovirus*, family *Geminiviridae*)

Ishtiaq Hassan[1,2,3], Anelise F. Orílio[1], Elvira Fiallo-Olivé[1], Rob W. Briddon[2] & Jesús Navas-Castillo[1]

[1]Instituto de Hortofruticultura Subtropical y Mediterránea "La Mayora", Universidad de Málaga - Consejo Superior de Investigaciones Científicas (IHSM-UMA-CSIC), Estación Experimental "La Mayora", 29750 Algarrobo-Costa, Málaga, Spain.
[2]Agricultural Biotechnology Division, National Institute for Biotechnology and Genetic Engineering, Faisalabad, Pakistan.
[3]Pakistan Institute of Engineering and Applied Sciences, Islamabad, Pakistan.

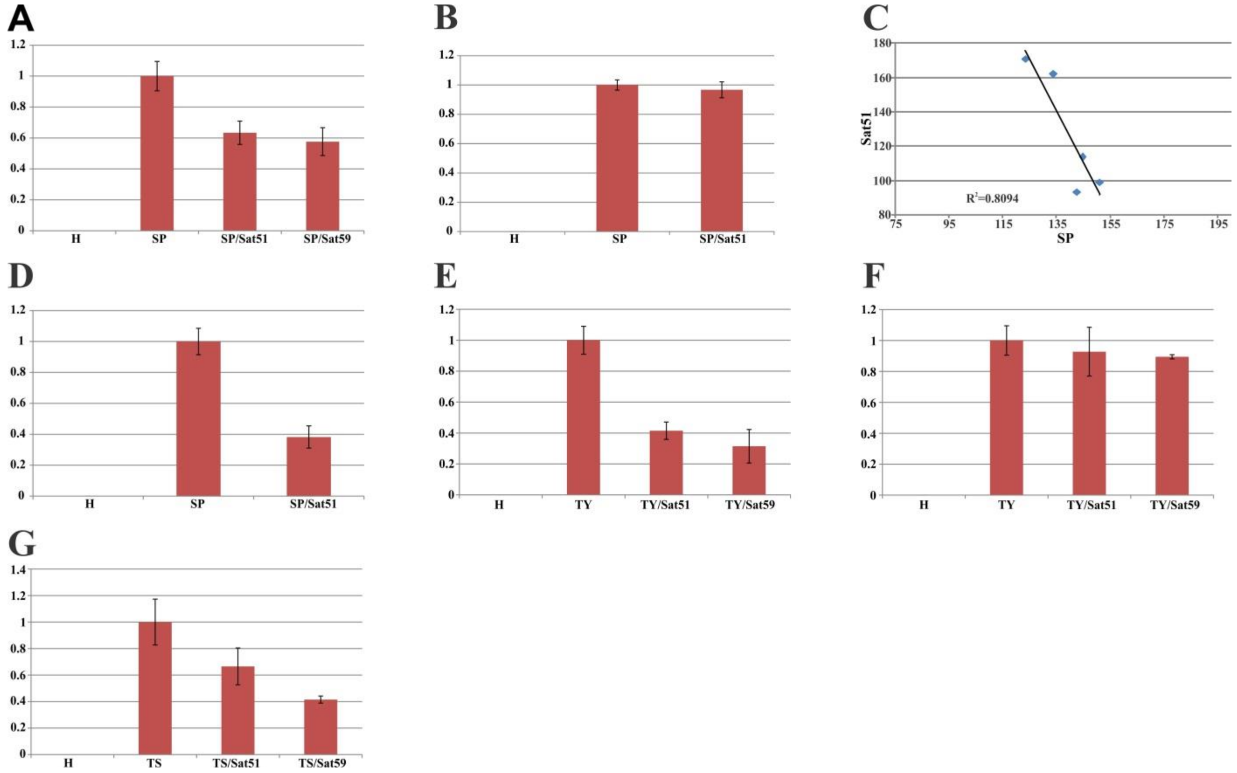

Supplementary Figure 1. ImagJ analysis of Southern blot band intensities.
The bar graphs show the mean value (with standard error of the mean) of calculated virus (either sweet potato leaf curl virus [SP], tomato yellow leaf curl virus [TY] or tomato yellow leaf curl Sardinia virus [TS]) levels (based on band intensities). The levels were calculated from the Southern blots in Fig. 2A **(A)**, Fig. 2B **(B)**, Fig. 2C **(D)**, Fig. 4A **(E)**, Fig. 4B **(F)** and Supplementary Fig. 2B **(G)**. In each case the levels of virus in co-infections with satellites SBG51 (Sat51) or SBG59 (Sat59) were calculated by taking the levels in plants infected with only virus as 1.0. **(C)** A scatter diagram showing the relationship between the calculated levels of SPLCV (SP) and SBG51 (Sat51) in SPLCV/SBG51 infected *I. setosa* plants calculated from the blots in Fig. 2B.

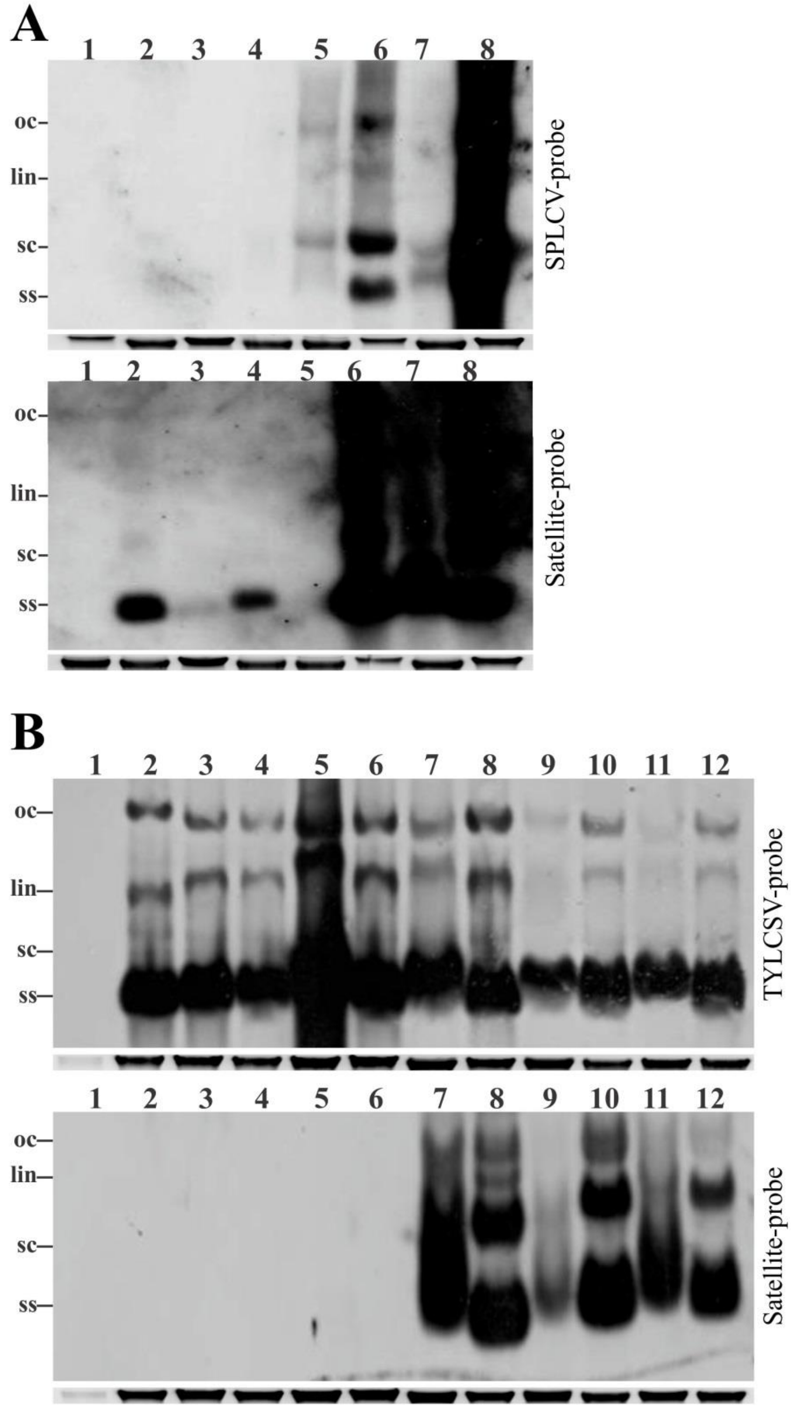

Supplementary Figure 2. Southern blot analysis of DNA samples extracted from begomovirus infected plants.

(A) The DNA extracts were from sweet potato cv. Beauregard (lanes 2-4), cv. Promesa (lanes 5-7) and *I. setosa* (lane 8) plants graft-inoculated with scions from SPLCV/SBG51 infected *I. setosa* plants. A DNA sample extracted from a healthy, non-inoculated sweet potato cv. Promesa plant was run as a control (lane 1). **(B)** DNA was extracted from *N. benthamiana* plants inoculated with TYLCSV (lanes 2-6), TYLCSV and DIM-SBG51 (lanes 7-9) or TYLCSV and DIM-SBG59 (lanes 10-12). The DNA extract run in lane 1 was from healthy non-inoculated *N. benthamiana* plant. Blots were probed for the presence of virus (upper panel) and SBG51 (lower panel) in each case. The positions of viral single stranded (ss) super-coiled (sc), linear (lin) and open-circular (oc) DNAs are indicated. The ethidium bromide-stained genomic DNA band on the gel is shown below the Southern blot in each case. DNA was extracted at 20 dpi and 6 µg of total DNA was loaded in each case.
